# Assessment of the organizational factors in incident management practices in healthcare: A tree augmented Naive Bayes model

**DOI:** 10.1371/journal.pone.0299485

**Published:** 2024-03-07

**Authors:** Salma Albreiki, Mecit Can Emre Simsekler, Abroon Qazi, Ali Bouabid

**Affiliations:** 1 Department of Management Science and Engineering, Khalifa University of Science and Technology, Abu Dhabi, United Arab Emirates; 2 School of Business Administration, American University Sharjah, Sharjah, United Arab Emirates; 3 Institute of Educational Sciences, Mohammed VI Polytechnic University, Ben Guerir, Morocco; National University of Modern Languages, PAKISTAN

## Abstract

Despite the exponential transformation occurring in the healthcare industry, operational failures pose significant challenges in the delivery of safe and efficient care. Incident management plays a crucial role in mitigating these challenges; however, it encounters limitations due to organizational factors within complex and dynamic healthcare systems. Further, there are limited studies examining the interdependencies and relative importance of these factors in the context of incident management practices. To address this gap, this study utilized aggregate-level hospital data to explore the influence of organizational factors on incident management practices. Employing a Bayesian Belief Network (BBN) structural learning algorithm, Tree Augmented Naive (TAN), this study assessed the probabilistic relationships, represented graphically, between organizational factors and incident management. Significantly, the model highlighted the critical roles of morale and staff engagement in influencing incident management practices within organizations. This study enhances our understanding of the importance of organizational factors in incident management, providing valuable insights for healthcare managers to effectively prioritize and allocate resources for continuous quality improvement efforts.

## 1. Introduction

The healthcare industry is experiencing rapid growth and significant transformation. However, the presence of operational failures and mismatch between supply and the increasing demand pose challenges to the provision of safe and effective care within complex healthcare systems. Such challenges dramatically increase the rate of medical errors, with a significant impact on patient safety. Medical errors rank as the third leading cause of death in the US following cancer and heart diseases [1]. According to a pioneering study conducted by the Institute of Medicine (IOM), an estimated 44,000 to 98,000 deaths occur in hospitals each year in the US due to preventable medical errors [2]. Similarly, in the UK, approximately 850,000 medical errors are reported annually, with nearly half of them being preventable [3]. These errors stem from various organizational factors within the dynamic healthcare systems [4].

While multiple factors may influence patient safety in hospitals [5], incident management stands out as a critical practice with a significant impact on learning from failures and mitigating potential risks. Numerous methods have been employed to identify the factors that affect incident management [6], emphasizing the need to assess the suitability of assessment methods and tools in healthcare organizations with the involvement of safety and measurement experts, whenever feasible [7]. Previous studies have also highlighted the limited application of statistical methods, which may have hindered the identification of complex associations between organizational factors and incident management practices [8]. Current measurement tools for incident management and patient safety primarily rely on surveys and questionnaires, with limited statistical models. Such approaches may not capture the complex relationships between multiple organizational factors and incident management practices, thus yielding limited results and hindering decision-making in healthcare management.

To address these limitations, data-driven Bayesian Belief Network (BBN) models can be potentially employed to explore statistical dependencies and causal probabilistic interactions among interconnected variables, providing graphical representations for enhanced understanding. In consideration of both the constraints and benefits associated with our research context, our research question is: “How can BBN models be utilized to determine and characterize the interactions and interdependencies among organizational factors and incident management practices?”

To address this research question, this study proposes a data-driven approach based on survey data to investigate the impact of various organizational factors on incident management practices. Data-driven models have gained considerable importance in supporting healthcare professionals in operations management and decision-making processes [9]. By leveraging the characteristics of BBNs, this study aims to move beyond simple data analysis to provide in-depth insights, probabilistic outcomes, and valuable conclusions. Ultimately, these findings aim to inform and facilitate the effective allocation and prioritization of resources, thereby enhancing incident management practices.

The paper’s outline is as follows: Section 2 provides a comprehensive review of the existing literature on incident management and organizational factors in patient safety context. Section 3 presents the method employed in this study. Section 4 presents the results, analysis, and interpretations. Section 5 discusses the implications of the research findings. Finally, Section 6 presents the key findings, contributions, study limitations, and directions for future research.

## 2. Literature review

### A. Incident management in healthcare

Patient safety has emerged as a significant global concern in recent decades. The rise in medical errors led healthcare organizations to progressively implement policies and practices aimed at enhancing patient safety. One key practice involves the prevention of incidents through the implementation of incident management systems designed to mitigate potential harm to patients [10]. Within healthcare settings, incident management includes measures taken by organizations to prepare for, respond to, and derive valuable insights from events or potential hazards [10, 11]. It represents a critical component in ensuring patient safety. Healthcare organizations can enhance patient safety and overall quality of care by establishing robust incident reporting systems, conducting thorough incident analyses, prioritizing efficient response protocols, and fostering a culture of continuous learning. Through the implementation of these measures, healthcare providers can effectively address incidents, extract meaningful lessons, and continuously improve their practices to deliver safer and higher-quality care [12].

Various incident management practices have been adopted to provide a collaborative strategy for incident reporting, investigation, and control in healthcare [13, 14]. These practices outline organizational processes and fundamental principles that facilitate efficient and effective incident management for all types of risks [15]. Incident reporting and investigation are integral aspects of effectively managing and responding to incidents [14]. Incident reporting plays a crucial role by providing vital information that shapes decision-making, resource allocation, analysis, and compliance. It enhances situational awareness, facilitates prompt and efficient responses, and fosters a culture of learning and improvement to better handle future incidents [16]. In the context of healthcare, incident reporting refers to a voluntary patient safety initiative where stakeholders involved in the patient care process, particularly nurses and physicians, provide comprehensive and in-depth information regarding medical errors, including near misses and unsafe conditions [17]. Near-miss events include events that are addressed before causing harm to a patient but had the potential to do so [18]. These events are more frequent than adverse incidents resulting in harm. Given their frequency and significance, increased attention to near-miss events may aid in the development of effective approaches to enhancing overall patient safety [19, 20]. For instance, ElKhider and Savage [21] identified incidents that could have become hazardous if not addressed in a timely manner, offering healthcare decision-makers an opportunity to examine root causes and develop solutions, thereby improving patient safety. Thus, healthcare organizations should consider near misses as opportunities to cultivate a culture of safety, which is instrumental in ensuring high-quality and safe patient care.

Incident management is widely acknowledged as an effective approach to enhancing patient safety. For an incident management system to be effective, healthcare organizations aim to establish a supportive environment, ensure the confidentiality of those reporting events, summarize reported incidents, disseminate analyses in a timely manner, and develop action plans. Thus, incident reporting encompasses the tracking, tracing, and reporting of adverse events, such as medication errors, that have the potential to harm patients or lead to devastating outcomes. In particular, incident reporting enables medical professionals to identify the nature and frequency of adverse occurrences in healthcare, facilitating the implementation of corrective measures, such as the establishment of policies to prevent recurrence [22, 23]. Additionally, studies show that incident reporting can lead to the introduction of staff training programs that raise awareness of risks and foster a culture of safety [24]. Training initiatives improve healthcare providers’ competencies, such as enhancing nurses’ drug administration skills, thereby enhancing patient safety [24]. However, despite these benefits, earlier studies argue that adverse events are significantly underreported or not reported at all. Studies attribute these outcomes to staff attitudes, concerns regarding confidentiality, and the absence of feedback after reporting incidents [25]. Consequently, the full benefits of incident reporting are not fully realized due to significant barriers that discourage reporting.

Understanding the number of incidents an organization typically encounters, how many are reported, and how many of the reported incidents undergo investigation is crucial. The incident reporting system serves as a means of bringing safety issues to the attention of management. Reporting incidents is an essential and mandatory aspect of the incident learning system, and maintaining a successful system requires adherence to specific steps, as depicted in the figure. These steps are designed to ensure that reported incidents receive proper support, that events are appropriately addressed, and that effective follow-up takes place, including the dissemination of lessons learned from the investigation of the incident, enabling departments to achieve tangible safety improvements.

The initial step involves identifying the incident, encompassing incidents that resulted in adverse events as well as those that had the potential to lead to adverse events, such as near misses, so that lessons can be learned and actions taken to prevent actual incidents from occurring in the future. The individual identifying the incident must take immediate action. Subsequently, the individual responsible for identifying the incident should complete a comprehensive report about it. The incident is then subject to investigation, which involves studying the facts surrounding the incident to uncover its root causes. The organization should then act upon the recommendations generated during the investigation stage by developing an implementation strategy and identifying the necessary resources. Finally, the lessons learned from the incident investigations should be shared with staff. The purpose of organizational learning is to foster an organizational culture wherein individuals do not need to experience an incident firsthand to learn from it.

The National Health Service (NHS) in the UK has a well-established incident reporting system known as the National Reporting and Learning System (NRLS). Established in 2003, the NRLS serves as a central database for patient safety incident reports, which are subsequently analyzed to identify hazards, risks, and opportunities for improving patient care safety [26]. Managed by NHS Improvement, the NRLS also provides advice and guidance to independent NHS organizations to minimize patient risks. Initially, the NRLS operated on a voluntary basis until 2010, when the reporting of serious incidents and deaths became mandatory [26]. Despite various efforts to raise awareness of incident reporting, it is expected that several organizational factors may influence reporting. For example, the NHS summarizes various organizational factors to provide an overview of staff experience. Equality, Diversity, and Inclusion entail fair treatment and equal opportunities for all individuals, regardless of personal characteristics such as religion, gender, ethnic background, age, sexual orientation, and disability [27]. It also encompasses creating a sense of belonging for all employees within the organization [28]. Health and wellbeing focus on the link between work, an employee’s health status, and the role of organizational stakeholders, including management, in implementing organization-wide strategies that promote staff health and wellbeing [29].

The immediate manager refers to the individual from whom a medical expert receives instructions, assignments, and work-related projects. This person is the closest member of an organization’s management and takes an interest in workers’ health, opinions, and work-related concerns [30]. Morale reflects the satisfaction, motivation, engagement, respect, recognition, and support that employees within a healthcare organization experience in the workplace. Additionally, morale evaluates the extent to which employees willingly contribute to achieving organizational objectives [31].

Quality of care pertains to the degree to which health services provided increase the likelihood of achieving targeted health outcomes. High-quality care is perceived as effective, safe, person-centered, timely, equitable, integrated, and efficient [32]. Safe environment bullying in the workplace refers to inappropriate behaviors or actions that psychologically, mentally, or physically harm healthcare employees. Bullying can take various forms, including excessive supervision from immediate managers, overly harsh or unjust criticism from supervisors, threats, verbal abuse, and physical assault [33].

Safe environment violence encompasses physical assault, harassment, and intimidation directed towards medical professionals in the workplace. Common forms of violence perpetrated against healthcare workers, such as nurses, include physical aggression, verbal abuse, and mobbing from patients and their families, as well as sexual harassment, discrimination, and intimidation from managers and colleagues [34–36]. Safety culture refers to shared beliefs, perceptions, and values among healthcare professionals regarding health and safety management. A safety culture in medical institutions is characterized by elements such as collaboration, organizational learning, effective communication, shared cultural perception, and the provision of constructive and non-punitive feedback and responses to medical errors [37].

Staff engagement in healthcare refers to the involvement of medical professionals in critical organizational activities, including decision-making and problem-solving, to the extent that they display enthusiasm and dedication towards contributing to the achievement of organizational goals [38]. Teamwork involves working collectively as a group to accomplish shared objectives [39]. Successful teamwork necessitates leadership, effective communication, training, well-defined team rules, and a clearly defined purpose.

In conclusion, patient safety has emerged as a growing concern worldwide in recent decades, prompting healthcare organizations to implement policies and changes aimed at enhancing it. Incident management plays a crucial role in ensuring patient safety by proactively preparing for, responding to, and gaining insights from events or potential hazards in healthcare settings. Incident management supplies vital information that shapes decision-making, resource allocation, analysis, and compliance. It enhances situational awareness, facilitates a prompt and efficient response, and fosters a culture of learning and improvement. Despite its numerous benefits, incident management faces significant barriers that hinder reporting and investigation efforts.

While organizational factors, mentioned above, may have an impact on incident management practices, there is limited evidence investigating their interactions and relative importance to the outcome. Further, the examination of potential interdependencies between organizational factors and incident management practices using probabilistic and graphical models has been lacking. To address this gap, the utilization of robust tools, such as BBN models, may provide significant opportunities through graphical representations of the interactions of the system with high prediction capabilities. In this research context, BBN models can effectively aid in predicting the relationships between organizational factors and incident management, while also identifying the relative importance of these factors. By employing BBN models, a comprehensive understanding of the intricate connections and dependencies within the organizational context can be gained, contributing to more informed decision-making and proactive measures in incident management.

### B. Bayesian belief network

A BBN is a probabilistic graphical model that presents a number of variables and their probabilistic relationships [40]. BBNs are represented as directed acyclic graphs (DAGs), which are widely used in the fields of statistics, machine learning, and artificial intelligence. A DAG is made up of a set of variables (nodes) and the relationships between them (arcs) [41]. Probability distributions capture the intensity of the relationships among interconnected variables. Any variable node’s probability distribution is determined only by its parents. As a result, the probability distribution in a Bayesian network (BN) with n nodes (X1, …, Xn) is represented in Eq (1):

$$P(X_{i})=\Pi_{i}^{n}P(X_{i}|P_{a}(X_{i}))$$ (1)

where *P_a_*(Xi) is the set of probability distributions corresponding to node Xi’s parents. BBNs are now recognized as an effective tool for risk analysis and decision support in real-world problems.

**Fig 1:**
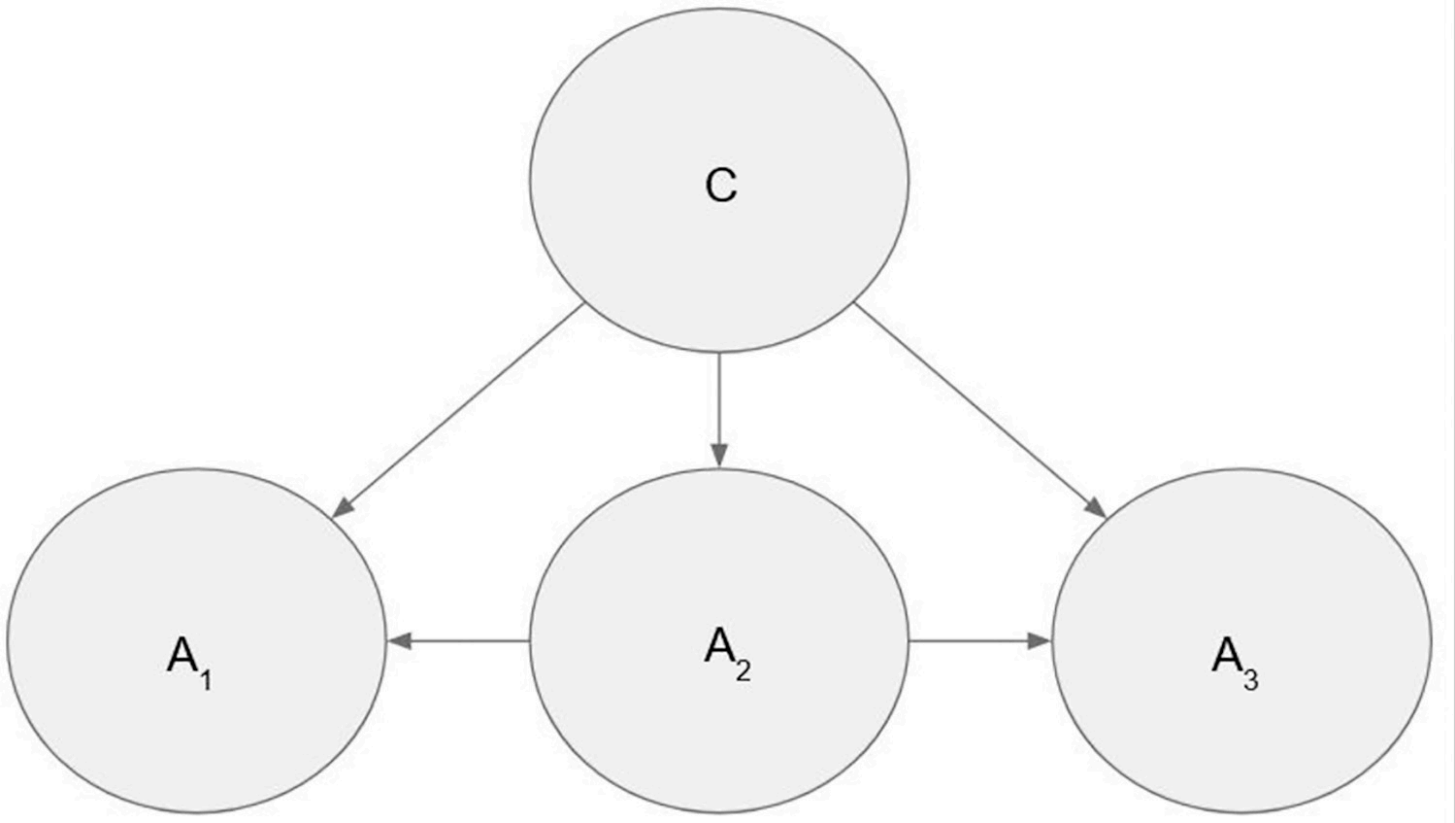
Tree augmented Naive Bayes structure.

Many different algorithms can be used to learn the structure of a Bayesian network [42, 43]. These algorithms are classified as constraint-based or score-based. The constraint-based approach involves constructing a network utilizing data by using conditional independence statements [44]. The score-based approach, on the contrary, involves the use of a scoring function to assess the quality of Bayesian network models and choose the one with the highest score [45]. Tree Augmented Naive Bayes (TAN), is one of the promising and commonly used structural learning algorithm with high prediction capabilities [46]. It is a semi-naive algorithm that is based on the Bayesian search method [47]. TAN is an enhancement on Naive Bayes that applies direct dependencies or interactions among attribute variables [48]. Fig 1 shows that the TAN networks does not only contain edges between the class node C and the attributes in A, but also between the individual attributes in A.

The TAN model allows for one level of interaction between random variables. The class node has a direct edge with all the feature variables. As a result, while computing the P(C|A_1_, A_2_, A_3_… A_n_), it will take all the variables into account. Furthermore, each variable is connected to another variable by a direct edge, except for the specialized property known as the root. TAN Bayes improves performance of the classifier as well as increasing the prediction accuracy while maintaining efficiency and model simplicity [49]. Because the interaction between the variables is limited to one, the computational complexity of this model is reduced. “Thus, TAN maintains the robustness and computational complexity of the Naive Bayes model while improving accuracy” [47].

BBNs have been successfully used in different domains and industries, such as healthcare [4, 50, 51], education [52], safety analysis [53], finance [54], risk management [55], disaster management [56], and traffic management [57]. BBNs account for noise in stochastic events, ensuring that strong interactions are highlighted across the data. They can also be used to identify causation, which makes them particularly useful, e.g., in understanding gene interactions [58]. Large datasets also exist within clinical practices, and BBNs can aid in understanding this data. For instance, the availability of large volumes of electronic patient records can help increase the accuracy of their risk assessments [59]. Historically, however, clinical analyses involved the use of regression-based models. In contrast, in a review, Arora highlighted the advantages of BBNs over regression-based models due to visually representing causal relationships between variables [60]. In another example, Aktas [61] used a BBN to create a decision support system (DSS) helping healthcare managers improve the efficiency of the resource allocation. BBNs can also be used to generate “what if” scenarios and aid in precision medicine by providing individual risk assessments [60]. Consequently, BBNs provide various opportunities in data analysis, ensuring the use of relevant and up-to-date practices in management and decision-making.

## 3. Methods

**Fig 2:**
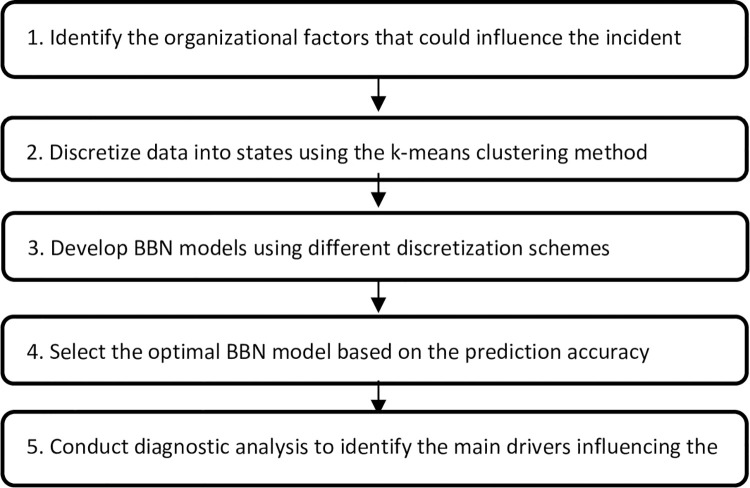
Research framework.

The research framework, assessing the relationships between various organizational factors and incident management, is presented in Fig 2. First, the organizational factors that can influence incident management practice were identified from the NHS survey data. The data were checked for missing values and then discretized into states using the k-means clustering method (using Python). Next, BBN models were created using various discretization schemes and the best BBN model, based on the prediction accuracy, was chosen for further analysis (using GeNIe 2.0 [62]). Next, diagnostic analysis, including sensitivity and scenario analysis, were implemented to identify how organizational factors influence incident management practice in different circumstances. These approaches assist decision-makers in ranking significant risk drivers and enable them to assess the relative importance of factors in successful resource allocation.

### A. Data collection

The UK NHS Staff Survey [63] data was used to capture various organizational factors and incident management practices. Over a million NHS staff have been encouraged to join and share their perspectives on their experiences working for their respective NHS organizations. The Staff Survey questions aim to ensure a complete understanding of working conditions across the NHS employers and national stakeholders concerning staff experience. To validate data quality, NHS England performs high-level validations on the data given by NHS trusts (known as organisational unit within the NHS) [64].

The dataset used in this research includes survey data from 2018 to 2020 that the research team accessed it from the beginning of February, 2022 for research purposes. The research team had no access to information that could identify individual participants during or after data collection. There are nine reporting themes in the data that might be relevant to patient safety: (X1) *Equality*, *diversity & inclusion*; (X2) *Health & wellbeing*; (X3) *Immediate managers*; (X4) *Morale*; (X5) *Quality of care*; (X6) *Safe environment–Bullying & harassment*; (X7) *Safe environment–Violence*; (X8) *Staff engagement*; (X9) *Team working*. The “Safety culture” theme was not considered because our outcome measure was one of the questions under the safety culture theme [64].

All themes are assessed on a 0–10 point scale and the mean scores are presented. A higher theme score always suggests a better outcome. Each theme addresses two to nine questions. Our outcome measure is also from the same survey data asking NHS staff their perception on the following, “*when errors*, *near misses or incidents are reported*, *the organization takes action to ensure that they do not happen again*” (X10).

### B. Data preprocessing

We utilized descriptive analysis to detect missing data and potential distributional outliers for the whole dataset prior to analysis. First, the NHS Staff Surveys across hospitals for the years between 2018 and 2020 were merged. Additional eligibility criteria were taken into account, focusing on hospital types to ensure consistency within the model. Consequently, only acute hospitals were incorporated, while other healthcare facilities, including ambulance and mental health hospitals, were excluded from the scope of this study. After removal of the missing data, 371 observations were considered to be analysed further. The goal of the descriptive analysis summarized in Table 1 shows the descriptive results of the data analysis.

**Table 1:** Descriptive analysis of data.

ID	Variable	Count	Mean	Median	SD	Min	Max	Range
X1	Diversity	371	9.03	9.10	0.29	8.06	9.63	1.57
X2	Health & wellbeing	371	5.95	5.95	0.28	5.19	6.87	1.69
X3	Immediate managers	371	6.81	6.81	0.21	6.20	7.49	1.29
X4	Quality of care	371	7.47	7.47	0.20	6.96	8.13	1.17
X5	Morale	371	6.16	6.17	0.25	5.42	6.90	1.47
X6	Staff engagement	371	7.03	7.03	0.23	6.39	7.65	1.26
X7	Safe Environment (SE)–Bullying & harassment	371	7.98	8.01	0.28	7.11	8.69	1.58
X8	Safe Environment (SE)–Violence	371	9.45	9.45	0.10	9.08	9.76	0.68
X9	Team working	371	6.56	6.55	0.22	5.91	7.31	1.40
X10	Incident Management	371	0.71	0.71	0.05	0.56	0.84	0.28

As shown in Table 2, an intercorrelation analysis of the organizational factors was also performed using the Spearman’s coefficient to examine the statistical characteristics and assess the strength of interaction between the factors.

**Table 2:** Intercorrelation using spearman’s coefficient.

ID	Variables	X1	X2	X3	X4	X5	X6	X7	X8	X9	X10
X1	Diversity	1									
X2	Health & wellbeing	0.56	1								
X3	Immediate Managers	0.48	0.70	1							
X4	Quality of care	0.04	0.39	0.48	1						
X5	Morale	0.69	0.86	0.80	0.42	1					
X6	Staff engagement	0.32	0.70	0.78	0.66	0.79	1				
X7	SE—Bulling & harassment	0.78	0.72	0.62	0.15	0.76	0.51	1			
X8	SE—Violence	0.32	0.25	0.22	0.02	0.30	0.21	0.42	1		
X9	Team working	0.35	0.52	0.82	0.53	0.64	0.75	0.43	0.17	1	
X10	Incident Management	0.32	0.65	0.65	0.60	0.69	0.78	0.57	0.29	0.54	1

Most BBN algorithms work with discrete data [65]. Therefore, the aggregate-level survey data was discretized before employing the TAN algorithm [46, 66]. The *k*-means clustering method was used to discretize the data before modeling it in this study (using Python Scikit-learn library) [67].

The *k*-means is a well-known and widely used clustering method [68]. It is useful for discretizing continuous variables because it computes a continuous distance-based similarity measure to cluster data points [69]. It originates from signal processing aimed at partitioning and observing *k* clusters in which each observation is the cluster that has the nearest mean, which serves as the cluster’s prototype [70]. The discretization strategy for input data occurs via the use of the maximum and minimum dataset values, computed cluster centers, and the midpoints between every two clusters. The silhouette analysis is used to determine the value of “K”. Some of the variables resulted in two clusters while the others had three as shown in Table 3. Therefore, in this study, we tested three discretization schemes to discretize the values for BBN modelling.

**Table 3:** The silhouette analysis results with optimum K value.

ID	Variable	K value
X1	Diversity	2
X2	Health & wellbeing	2
X3	Immediate managers	3
X4	Quality of care	2
X5	Morale	2
X6	Staff engagement	3
X7	SE–Bullying & harassment	2
X8	SE–Violence	3
X9	Team working	3
X10	Incident Management	3

### C. Data modeling and analysis

In this stage, BBN models were created adopting the discretization scheme described in the previous section (using GeNIe). The BBN models were verified using a k-fold cross-validation approach [71], which consists of dividing a dataset into *k* equal-sized parts, training the network on *k*-1 parts, and testing it on the final *k*th part [72]. The procedure is then repeated *k* times, with each testing iteration using a new part of the data [73]. There are differing views on the best value of *k* [71]; a value that is too low might lead to biased findings while a value that is too high can lead to excessive computing times. A value of *k* = 10 is generally seen as reasonable [74]. The validation result demonstrates the class node accuracy in testing the efficacies for various discretization schemes. Eq (2) can be used to calculate the prediction accuracy of such data-driven models.


$$Accuracy=\frac{number\;of\;correct\;predictions}{total\;number\;of\;predictions}$$ (2)

## 4. Results

### A. BBN model analysis

Considering different discretization schemes, we produced three different BBN models. The models’ prediction accuracy was then evaluated using a 10-fold cross-validation procedure. Table 4, shows that the two states discretization scheme had the highest prediction accuracy of 78.7%. As a result, the remaining procedure was implemented in a two-state discretization scheme using the TAN algorithm.

**Table 4:** Prediction accuracy of models with different discretization schemes.

Discretization Scheme	Prediction Accuracy
(Number of States)	(In Percentage)
Two states	**78.7**
Three states	70.0
Mix states	69.0

The confusion matrix in Table 5 illustrates the relationship between the TAN algorithm’s actual and predicted states. The bold numbers represent the numbers of accurately detected predictions for the class node. In the validation stage, out of 371 records tested, 292 were correctly identified with an accuracy of 78.7%.

**Table 5:** Confusion matrix with two-state discretization scheme.

		Predicted	
		State 0	State 1
Actual	State 0	**136**	32
	State 1	47	**156**

**Fig 3:**
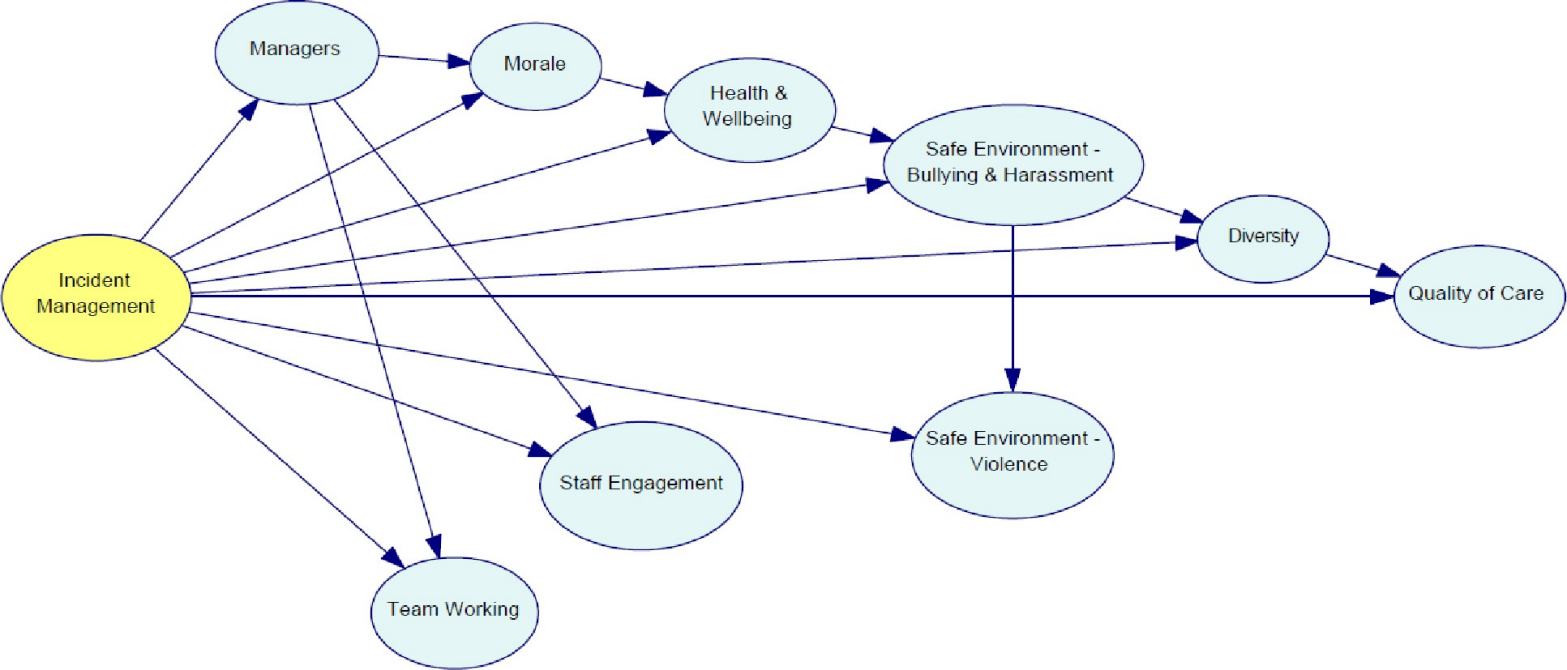
Network structure developed using TAN algorithm.

Using the TAN algorithm, Fig 3 shows the arcs between the 10 variables. This model illustrates statistical interdependence among variables regarding the dataset used in this research. All variables are dependent variables and each one has a direct relationship between the class node and one extra variable, except for managers. Managers is the only independent variable that only depends on incident management practice. This node is called the “root” (i.e., a node without any parent).

**Fig 4:**
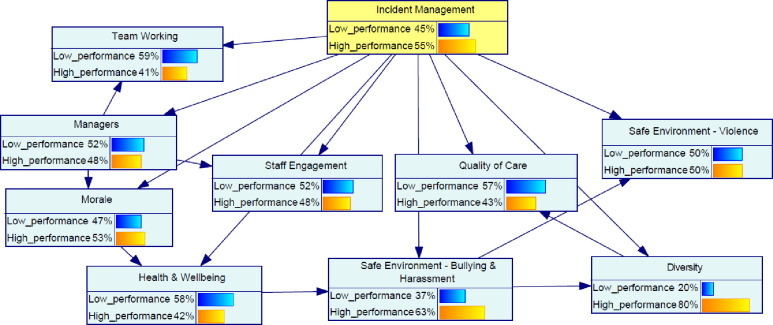
Probability distribution of factors associated with incident management.

The probability distribution of the organizational factors associated with the incident management practice is displayed as a bar chart in Fig 4. According to this model, 55% of the cases were related to State 1 (high state) of incident management practice, while 45% of the cases were related to State 0 (low state). Diversity had the highest probability of State 1 (80%) among all the factors considered in the model.

**Fig 5:**
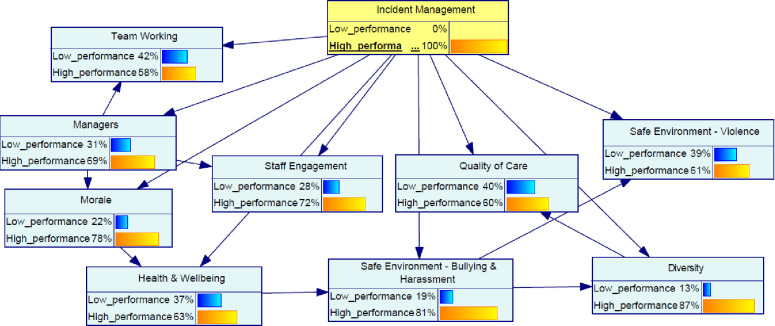
Effect on variable once the high state incident management is established.

The model in Fig 4 was then examined for the high state (see Fig 5) of the incident management to evaluate the change across variables in the network.

**Fig 6:**
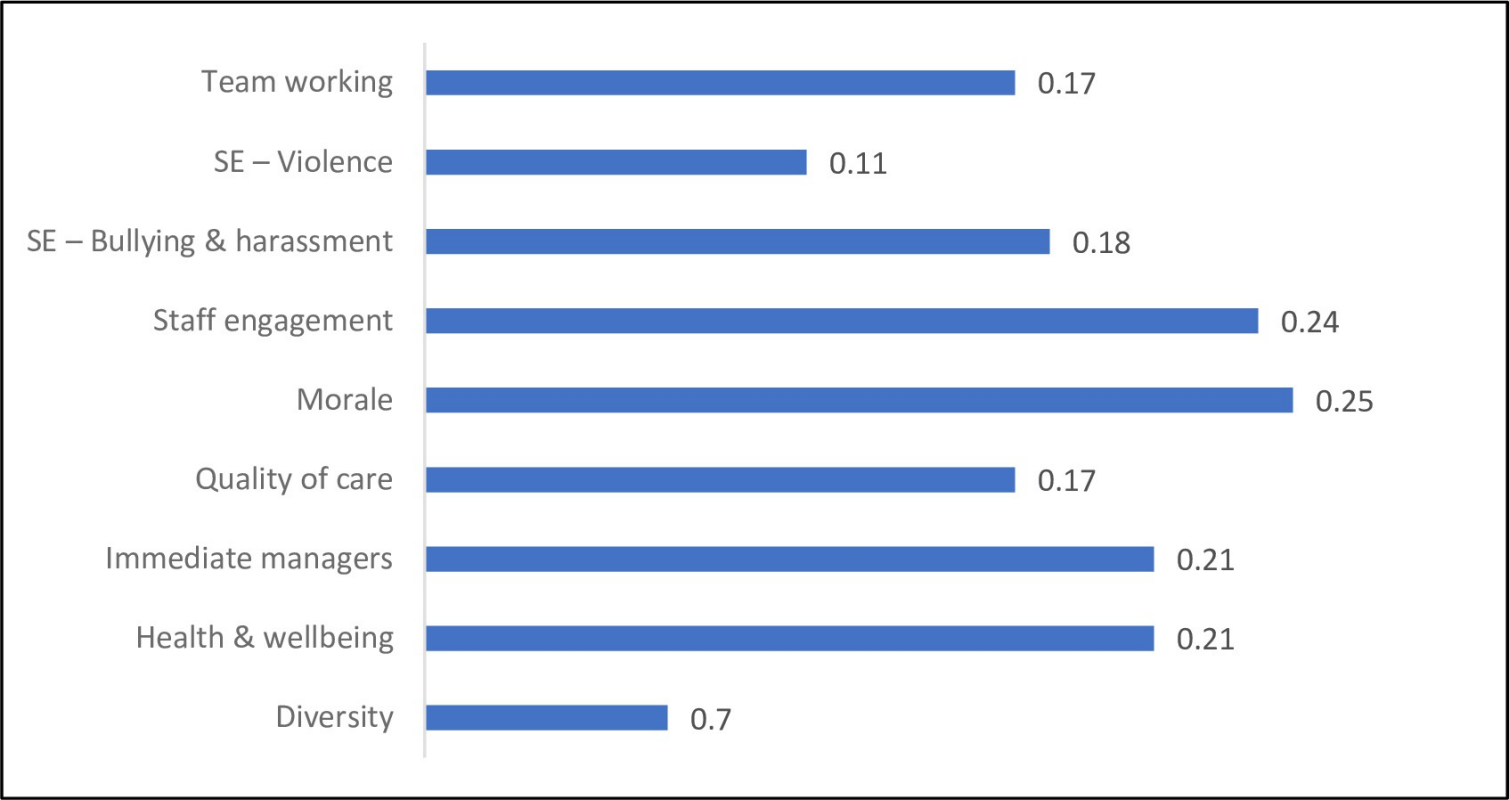
Back propagation impact assessment given the incident management in the high state.

Furthermore, the impact assessment of the organizational factors given the high state of incident management is summarized in Fig 6. The figure shows the increase in the probability of State 1 (as a percentage) for each variable. This assessment presents how much improvement is required across different variables to be able to optimize the target variable. Overall, the results showed that morale and staff engagement are the two leading factors in the backpropagation assessment given a high state of incident management.

### B. Diagnostic analysis

**Fig 7:**
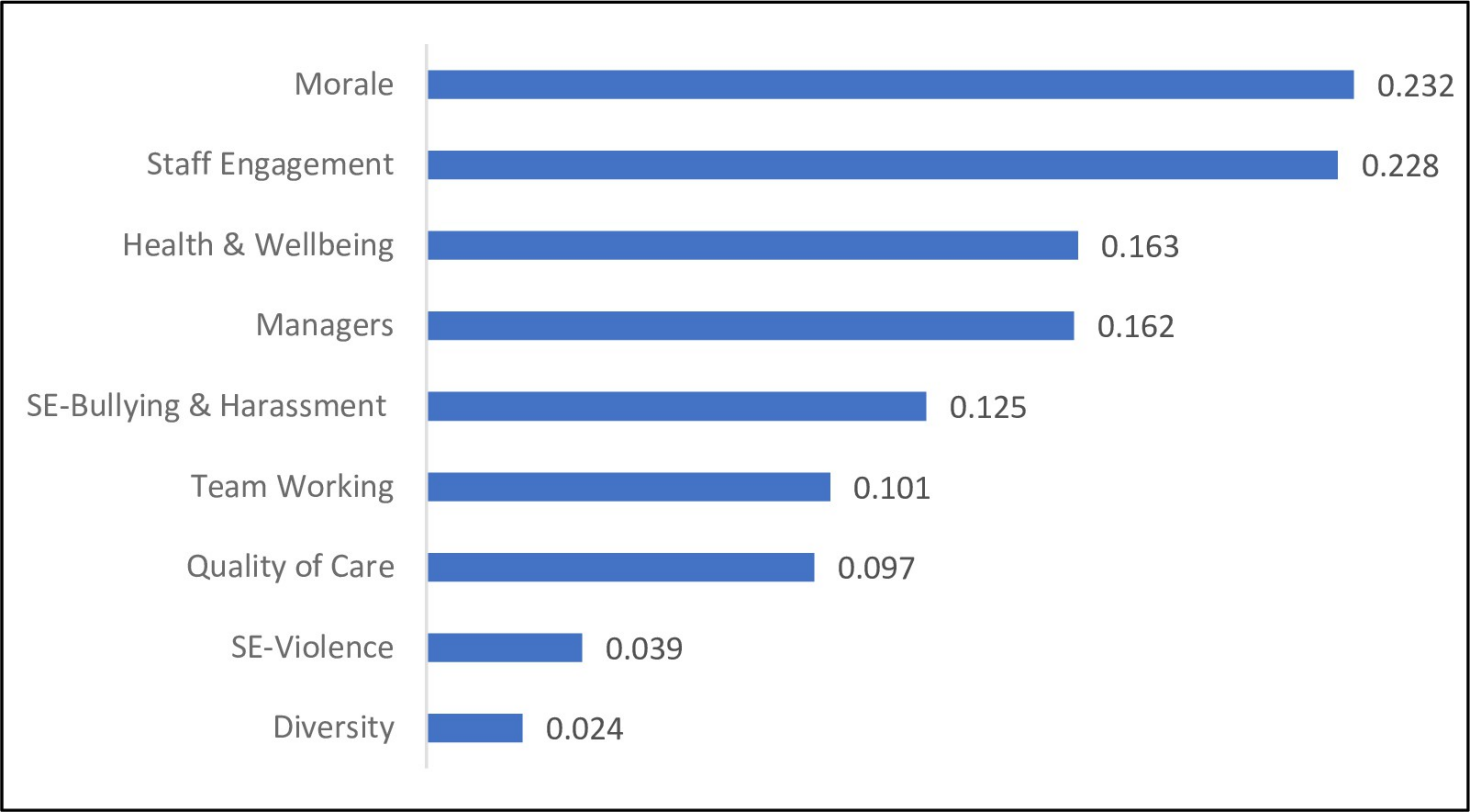
Diagnostic values of the organizational factors.

Diagnostic analysis is a feature that enables users to understand the factors and their relative importance in influencing incident management practices. The relative measure is based on cross-entropy, an information-theoretic measure that reflects the predicted reduction in entropy of the probability distribution across the target variable after viewing each domain separately. Cross-entropy is a utility-free measure of information value that provides an accurate evaluation of the value of the data in diagnosing the disorder in question [72]. Fig 7 shows the observations ranked from the most to the least informative. Morale was identified as the most influential domain because it yielded the highest diagnostic value (0.232), and staff engagement was identified as the second-most influential domain with a value of (0.228). This suggests that risk managers and decision makers can prioritize promoting morale and staff engagement to gain substantial benefits from the incident management practice.

## 5. Discussion

There are many organizational factors that may affect incident management practice. Utilizing the BBN model, the results showed that all nine factors included in this study have an impact on incident management practice. Further, the model explored the factors and identify their interdependencies and relative impacts on incident management practice. The most influential drivers in our models were found to be the morale and staff engagement factors, which were identified using diagnostic analysis. These factors have been recognized in the literature as factors that contribute to safety risks in a variety of areas, including healthcare. The morale of healthcare professionals is widely perceived as an important factor in safety and quality of care.

According to Sabitova et al. [75], healthcare providers with positive job morale are more likely than others to deliver high-quality care to patients. In addition, they link job morale with enhanced job performance and improved retention among medical professionals [75]. Similarly, Sania and colleagues [31] argued that workers with positive morale were happy, productive, creative, satisfied with their jobs, and committed to attaining organizational objectives instead of personal goals. Furthermore, in a study on the relationship between patient safety and incident management, Kelly et al. [76] identified a link between employee optimism and patient experiences. They showed that employees with positive morale shared effective practices, inspired and facilitated quality improvement, valued safe prescribing practices, and supported innovations. Notably, these outcomes improve patient safety. Thus, improved morale benefits healthcare organizations by enhancing staff performance, employee retention, and patient safety.

There exists a positive correlation between near misses and incident management and morale. For example, Kelly and colleagues [76] suggested a new approach to incident management that enabled healthcare organizations to recognize and capture learning from events of peer-reported excellence. Upon evaluating the proposed system—the learning for excellence model—the authors identified that it positively influenced team learning, patient care, and staff morale. Arguably, managing near misses and incidents helps capture valuable workarounds and adaptations, promotes excellence in practice, and advances staff competencies through medical training and professional development, leading to improved morale.

Successful healthcare leaders are aware of the organizational benefits of staff engagement. According to Kruse [77], employee engagement increases people’s emotional commitment to an organization. For example, it was shown that engaged workers are more likely to care about their institutions, colleagues, and patients than their disengaged counterparts [77]. In addition, Vidal [78] argued that engagement makes employees feel worthwhile and useful, motivating them to infuse empathy into their clinical care and invest their mental, physical, and emotional energies into job performance. Moreover, Vidal [78] reported that engaged staff are attentive to details and connected to organizational missions, purposes, and people. As a result, they are committed to meaningfully contributing toward improving job performance, which encompasses patient safety outcomes. Hence, staff engagement increases staff commitment toward attaining organizational goals and objectives, including patient safety. Employees feel more engaged and more innovative in their work when their organization reacts to their complaints [79].

Staff engagement directly impacts near-miss reporting and managing incidents. For example, Ashcroft [80] reported that employees who were less engaged with incident management systems were less likely to report adverse events than others because they felt that reporting was statistically insignificant. In addition, Macrae [81] argued that the lack of staff engagement could make employees perceive incident management as simply logging problems and waiting for solutions instead of an opportunity for learning and sharing insights. Furthermore, organizations that do not recognize the significance of staff engagement may appear to provide insufficient, meaningless, or no feedback to workers after reporting adverse events [81]. Notably, feedback is instrumental in demonstrating the value of incident reporting and informing people about actions taken and lessons learned. Thus, the lack of staff engagement impedes near-miss and managing incidents.

According to our findings, healthcare organizations should support better staff experience to prevent incidents and near misses that may harm patients and staff. Although all nine themes had statistically significant relations with incident management, the results also presented important insights and suggestions on the relative impact of the factors. As a result, morale and staff engagement may require additional attention for enhanced safety initiatives to be realized.

While moral and staff engagement emerge as relatively more important factors in incident management practices, it is crucial to recognize that all identified factors are linked to the effectiveness of incident management. While optimizing resources and prioritizing efforts towards these key factors can yield significant benefits, it is equally important to understand the root causes of organizational factors. For instance, past studies have indicated that low reporting rates may stem from issues such as the perceived lack of usefulness in reporting, time constraints in busy work environments with competing priorities [82], blame culture [83] and lack of feedback [84]. To enhance our understanding, further research could also explore whether specific groups of healthcare professionals (e.g., nurses and physicians) or certain types of incidents (e.g., medication errors) are more prone to identification and management in healthcare settings. Conducting such analyses may offer deeper insights into effective approaches for improving incident management practices.

## 6. Conclusions

### A. Contributions and practical implications

In healthcare, there has been a remarkable surge in growth and substantial transformation in recent years. Nevertheless, the existence of operational failures and a lack of synchrony between supply and the surging demand present formidable obstacles to ensuring the safe and efficient delivery of care. To prevent such challenges, incident management plays an important role to learn from the errors and experiences; however, this practice also has major limitations to be effective within complex and dynamic healthcare systems. This study presents a comprehensive framework aimed at understanding and mapping the causes of incident management practices by identifying the key organizational factors. Using a BBN approach, it underscores the significance of incident management practices while highlighting a gap in the current literature regarding the most influential factors and their interactions with other organizational aspects. The main contribution of this study lies in the identification of the key factors influencing incident management practices through the application of a data-driven BBN with the enhanced graphical model.

The findings of this study offer practical implications for decision-makers, enabling them to allocate resources effectively to enhance incident management practices. The diagnostic analysis revealed that morale and staff engagement emerged as the leading factors influencing incident management practices. These results provide hospitals with crucial insights into the organizational factors that significantly influence incident management. Additionally, our findings can assist healthcare organizations in prioritizing resources and addressing specific aspects causing challenges. By identifying eight interdependencies and recognizing the sole dependency of the *managers* variable on incident management practice, this study uncovers hidden information regarding nonlinear relationships between factors and highlights the importance of exploring interactions among the organizational factors.

This research contributes to the advancement of incident management practices by providing a comprehensive framework and valuable insights into the interplay of organizational factors. The outcomes support evidence-based decision-making, enabling healthcare institutions to optimize their incident management strategies for patient safety enhancement.

### B. Limitations and future work

While this study contributes valuable insights, it is important to acknowledge its limitations. Firstly, the data had to undergo discretization prior to applying the BBN, which may have impacted the prediction accuracy. Additionally, missing data is a common concern in medical field surveys, and this study is not exempt from that issue. Furthermore, as this research relies on hospital-level aggregate data, the relative importance rankings of organizational factors may vary among individual hospitals. Consequently, caution should be exercised in generalizing and transferring the findings to other countries, as the relationships between staff experience, errors impacting patient and staff safety, and organizational factors may differ.

Future research can address these limitations and expand upon this study in several ways. Firstly, incorporating additional factors and features that may influence healthcare operational failures would provide a more comprehensive understanding. Conducting the same methodology within a specific organization would enable researchers to delve deeper into its unique dynamics. Moreover, evaluating alternative discretization methods or machine learning approaches would allow for a comparison of prediction capabilities and potential variations in the relative importance of organizational aspects. Additionally, collecting data directly from patients would offer valuable insights into how their experiences align with operational failures and safety outcomes.

By addressing these considerations, future studies can enhance our understanding of the complex interplay between organizational factors, patient experiences, operational failures, and safety outcomes in healthcare settings.
